# Gross haematuria in the era of anticoagulant therapy – Implications on treatment and diagnostic approaches in a large emergency department patient population

**DOI:** 10.1002/bco2.70099

**Published:** 2025-10-08

**Authors:** Yushan Yang, Johanna Seidl, Simon Udo Engelmann, Maximilian Haas, Roman Mayr, Maximilian Burger, Johannes Breyer, Markus Resch

**Affiliations:** ^1^ Department of Urology University of Regensburg, Caritas St. Josef Hospital Regensburg Germany; ^2^ Department of Urology University Hospital Augsburg Augsburg Germany; ^3^ Department of Internal Medicine II University Hospital Regensburg, Caritas St. Josef Hospital Regensburg Germany

**Keywords:** anticoagulants, anticoagulation‐related haematuria, antithrombotic therapy, gross haematuria, urologic malignancy

## Abstract

**Introduction:**

Treatment with anticoagulants or antiplatelet drugs can provoke gross haematuria. In some cases, this may demask urologic malignancies. The goal of this study was to determine the influence of anticoagulants and antiplatelet drugs on the diagnosis and therapy of patients with gross haematuria who presented in the emergency department.

**Methods:**

This retrospective study analysed patients presenting with gross haematuria between January 1st, 2021 and December 31st, 2021 in a single centre university hospital. Information on pre‐existing conditions, anticoagulant and antiplatelet medication, and the further diagnostic and treatment course was gathered with a follow‐up time until December 31st, 2022.

**Results:**

Nearly half of the 541 patients (49.5%) presenting with gross haematuria were taking anticoagulant or antiplatelet medication. Patients receiving these medications were more likely to need bladder irrigation (p < 0.001). They were also more likely to be hospitalized (p < 0.001) and receive operative intervention (p = 0.011). The most common cause for haematuria was malignant tumours. A malignant urologic disease was diagnosed in 27% of the patients. Among those who were diagnosed with a malignant disease, the number of patients taking anticoagulant medication was higher (p = 0.005). In a follow‐up of 3 months, no thromboembolic events were observed after stopping or pausing anticoagulation or antiplatelet treatment.

**Conclusion:**

Clinically significant gross haematuria is often associated with the intake of anticoagulant or antiplatelet medication and can unmask underlying malignant diseases. The intake of anticoagulation or antiplatelet therapy should not be a reason to postpone diagnostic and therapeutic measures.

## 1 BACKGROUND

Gross haematuria is defined as visible blood in the urine. The differential diagnoses include urological and nephrological diseases and conditions. Causes for gross haematuria include benign and malignant diseases.^1^, ^2^

Direct oral anticoagulants (DOACs) are prescribed to reduce the risk of stroke or systemic embolism in patients with atrial fibrillation and to treat venous thromboembolism. Antiplatelet agents are widely used in primary and secondary prevention of myocardial infarction and ischemic stroke. Due to an increasingly ageing population with cardiovascular comorbidities, these medications have been encountered more frequently in urologic patients. The worldwide proportion of DOAC‐eligible patients using DOACs has increased from 42% in 2010 to 78% in 2018.^3^ In 2018, the overall prevalence of DOAC use among individuals aged over 40 in the United Kingdom was 4.9%, while the prevalence of antiplatelet therapy in the same age group was 11.6%.^4^

The intake of anticoagulants or antiplatelets can lead to complications such as intracranial haemorrhage or gastrointestinal bleeding. Especially the combination of anticoagulant and antiplatelet drugs increases the rates of haematuria‐related complications, emergency department visits and hospitalizations.^2^ Drug–drug interactions should also be taken into account, as other medications can affect the efficacy of DOACs or antiplatelet drugs, potentially influencing the severity of gross haematuria.^5^, ^6^ Switching and bridging refer to different strategies for managing bleeding complications while minimizing the risk of thrombosis. Specifically, switching implies replacing a DOAC with low molecular weight heparin (LMWH), whereas the term bridging refers to replacing a vitamin K antagonist with LMWH.^7^, ^8^

Previous studies have suggested that gross haematuria might unmask underlying malignant diseases in patients taking anticoagulants or antiplatelets.^9^, ^10^, ^11^ The incidence of diagnosing a malignancy in the urinary tract in patients with gross haematuria ranges around 20%.^12^ Patients with microhaematuria are significantly less likely to be diagnosed with a malignant disease compared to those with gross haematuria; for example, the IDENTIFY study reported malignancy rates of 6.38% for non‐visible haematuria versus 26% for visible haematuria.^1^, ^13^

The aim of this study was to examine the clinical significance of gross haematuria in patients receiving anticoagulant and antiplatelet therapy who present in the emergency department. The study examined whether patients receiving anticoagulation or antiplatelet therapy had higher rates of hospital admission, experienced significant blood loss, and whether the use of these medications influenced the diagnosis of malignancies. Furthermore, the objective was to examine whether the intake of those medications could influence early diagnosis of malignant urological diseases and if pausing the medication possibly led to the delay of diagnostic evaluation and therapy.

## 2 METHODS

This study included all patients who presented with gross haematuria between January 1st, 2021 and December 31st, 2021, in the emergency department of a single‐centre university hospital. Information on intake of anticoagulation or antiplatelet therapy was gathered by analysing the medical history, which was documented in a prospective manner at the time of presentation. Information on the further course, including discontinuation or pausing of the medication and further diagnostic steps, was collected using the electronic health record, including operative reports. Follow‐up for all patients was continued for one year until December 31st, 2022.

The study was approved by the University of Regensburg Local Ethics Committee (ethic vote 25–4286‐104). Written consent was not required, since all data were collected in a retrospective manner as part of routine diagnosis and treatment and analysed anonymously.

Statistical analysis was performed using SPSS version 29.0 (IBM, Armonk, NY, USA). An alpha value below 0.05 indicated statistical significance, and all analyses were considered two‐tailed. Continuous variables were reported as median and interquartile range (IQR). Categorical variables were evaluated using χ2 and Fisher's exact tests.

## 3 RESULTS

The population included 541 patients who presented with gross haematuria. The majority (78.4%) of the patients were male. The median age was 71 years (IQR 58–81) (Table 1). Smoking status was documented for 69 patients (12.7%). Among these, 22 were non‐smokers, 18 were active smokers and 29 had stopped smoking.

**TABLE 1:** Baseline characteristics *Hb = Haemoglobin in g/dl.*

	Total n = 541	Anticoagulants n = 268	No anticoagulants n = 273	p‐value
Age in years (IQR)	71 (58–81)	78 (70–83)	60 (48–74)	<0.001
Sex				<0.001
Male n (%)	424 (78.4)	227 (84.7)	197 (72.2)	
Female n (%)	117 (21.6)	41 (15.3)	76 (27.8)	
Irrigation n (%)	244 (45.1)	166 (61.9)	78 (28.6)	<0.001
Tamponade n (%)	83 (15.3)	56 (20.9)	27 (9.9)	<0.001
Hb (IQR)	13 (11.2–14.6)	12.4 (10.5–14.1)	14 (12.5–15)	<0.001
Transfusion n (%)	23 (4.3)	14 (5.2)	9 (3.3)	0.267
Inpatient admission n (%)	196 (35.7)	121 (45.1)	75 (27.5)	<0.001
Infection n (%)	191 (35.3)	89 (33.2)	102 (37.4)	0.312
Known malignant urologic disease n (%)	148 (27.4)	101 (37.7)	47 (17.2)	<0.001
Tumour diagnosed in further course n (%)	120 (22.2)	73 (27.2)	47 (17.2)	0.005
Readmission for surgery n (%)	214 (39.6)	110 (41)	104 (38.1)	0.483

Nearly half of the population (49.5%) received one or more types of anticoagulation or antiplatelet medication, including DOACs, antiplatelet agents, vitamin K antagonists or heparins. Most of the patients taking medication had one DOAC or one antiplatelet agent. Combinations of multiple medications were less common. The most common reason for antiplatelet therapy was vascular diseases such as coronary or peripheral artery disease; the most common reason for anticoagulation was atrial fibrillation. Of the patients with anticoagulant or antiplatelet medication, 6.7% had two or more diseases requiring anticoagulation. Detailed information on the frequencies of the medications and the reasons for their use is depicted in Table 2.

**TABLE 2:** Information on types of medications taken and pausing of medication, *low‐molecular‐weight heparin = LMWH, direct oral anticoagulants = DOAC.*

Parameter		n (%)
Type of medication (n = 268)	DOAC	100 (37.1)
	One antiplatelet agent	96 (35.8)
	Two antiplatelet agents	19 (7.1)
	Vitamin K antagonist	19 (7.1)
	LMWH	12 (4.5)
	Combination antiplatelet agent and DOAC	11 (4.1)
	Combination antiplatelet agent and LMWH	9 (3.4)
	Combination antiplatelet agent and vitamin K antagonist	1 (0.4)
	Combination LMWH and DOAC	1 (0.4)
Reason for medication (n = 268)	Chronic vascular disease	70 (35.1)
	Atrial fibrillation	54 (20.1)
	Multiple reasons	36 (13.4)
	Thromboembolic events	15 (5.6)
	Unknown	65 (24.2)
Medication discontinued (n = 268)	Continued	203 (75.7)
	Paused/discontinued	65 (24.3)
Duration of discontinuation (n = 65)	1 week	37 (56.9)
	2 weeks	22 (33.8)
	3 weeks	5 (7.7)
	4 weeks	1 (1.5)
Switching or bridging of medication (n = 65)	No	15 (23.1)
	Yes	50 (76.9)
Thromboembolic events during discontinuation of medication (n = 65)	None	65 (100)
Thromboembolic events after resuming medication (n = 65)	None	65 (100)

The median age of patients receiving anticoagulant or antiplatelet therapy was significantly higher than that of patients without such medication (78 years vs. 60 years, p < 0.001). The number of patients requiring bladder irrigation and hospitalization was also significantly higher (61.9% vs. 28.6%, p < 0.001). Patients treated with anticoagulants or antiplatelet agents had lower haemoglobin levels at the time of presentation (12.4 g/dl vs. 14 g/dl, p < 0.001) (Table 1). Both patients on antiplatelet monotherapy and those receiving dual antiplatelet therapy exhibited a significantly higher rate of gross haematuria compared to patients not receiving any medication.

The most common cause for gross haematuria was malignant tumours (Table 3). Tumours were diagnosed in 120 patients (22.2%). Infections were the second most common cause (19.8%). Recent urologic surgery represented a small portion (4.3%). Patients taking DOACs were more likely to have had a urologic operation in their medical history (p = 0.007) (Table 4). In 45 (8.3%) patients, no specific cause was found; of those patients, 25 (55%) were on anticoagulant or antiplatelet treatment.

**TABLE 3:** Cause of gross haematuria.

Cause	n (%)
Tumour	120 (22.2)
Infection	107 (19.8)
Urolithiasis	77 (14.2)
Catheter‐associated	69 (12.8)
Other	44 (8.1)
Benign prostatic hyperplasia	39 (7.2)
Medication‐associated	25 (4.6)
Prior urologic surgery	23 (4.3)
Unknown	20 (3.7)
Bladder Pain Syndrome	9 (1.7)
Radiation	5 (0.9)
Trauma	3 (0.6)

**TABLE 4:** Comparison of direct oral anticoagulants (DOACs) and no medication and of antiplatelet agents and no medication; *Hb = Haemoglobin in g/dl.*

	DOAC (n = 100)	No medication (n = 273)	p‐value	Antiplatelet agents (n = 115)	No medication (n = 273)	p‐value
Age (IQR)	78 (70–85)	60 (48–74)	<0.001	78 (67–82)	60 (48–74)	<0.001
Male n(%)	80 (80)	197 (72.2)	0.125	102 (88.7)	197 (72.2)	<0.001
Irrigation n(%)	50 (50)	78 (28.6)	<0.001	77 (67)	78 (28.6)	<0.001
Hb (IQR)	12.4 (10.7–13.9)	14 (12.5–15)	<0.001	13.2 (10.8–14.6)	14 (12.5–15)	<0.001
Transfusion n(%)	5 (5)	9 (3.3)	0.033	6 (5.2)	9 (3.3)	0.54
Inpatient admission n(%)	42 (42)	75 (27.5)	0.007	52 (45.2)	75 (27.5)	<0.001
Tumour diagnosed in further course n(%)	19 (19)	47 (17.2)	0.689	42 (36.5)	47 (17.2)	<0.001
urologic surgery in recent history n(%)	9 (9)	5 (1.8)	0.007	4 (3.5)	5 (1.8)	0.001

In 203 patients (75.7%), the medication was continued. Out of the 65 patients who paused the medication, switching or bridging was performed in 50 cases (76.9%) (Table 2). Patients for whom anticoagulation or antiplatelet medication was paused were older than those for whom medication was continued (81 years vs. 77 years, p = 0.03). No complications were recorded in a follow‐up of three months, neither during the medication pause nor after it was resumed.

It was more common for patients with anticoagulant or antiplatelet medications to have a malignant urologic disease in their medical history (37.7% vs. 17.2%, p < 0.001). Overall, patients taking medications were more frequently diagnosed with tumours compared to those without taking these medications (27.2% vs. 17.2%, p = 0.005). This was especially true for patients on antiplatelet agents (Table 4).

## 4 DISCUSSION

In our data analysis, we confirmed that gross haematuria can reveal urological conditions. Patients on anticoagulants or antiplatelet drugs were diagnosed with tumours more frequently than those without these medications.

Comparative analysis of more recent data with findings from earlier studies presents notable challenges, particularly due to the evolving landscape of anticoagulant therapy. In the past, a larger proportion of patients were prescribed vitamin K antagonists, whereas recent years have witnessed a significant shift toward the use of DOACs.^14^

In an analysis by Lawaczek et al. of 189 hospitalized patients with gross haematuria, 30.7% of patients had a pre‐existing genitourinary malignancy, and the occurrence of gross haematuria led to incidental diagnosis of a malignant disease in 6.4%. They also examined the duration and volume of irrigation, which we did not assess, and found that the intake of DOACs was linked to longer irrigation duration and greater irrigation volume.^5^

Similarly, an analysis by Mladenov et al. of 215 patients with gross haematuria showed urological pathology in most patients, with 33% being malignant diseases.^15^

In a retrospective observational study involving over 20,000 patients with haematuria over a decade, Yu et al. found that warfarin was linked to a higher prevalence and earlier detection of genitourinary cancer. Notably, patients on DOACs were not included in the study. The research revealed no significant difference in the incidence of stones and infections between patients taking anticoagulants and those who were not. Interestingly, within the group of patients diagnosed with bladder cancer, a greater number of low‐grade tumours were identified in patients on warfarin, suggesting that the medication may have increased bleeding in these individuals, leading to the earlier diagnosis of bladder cancer, which might have otherwise gone undetected for longer.^10^

In our cohort, patients receiving anticoagulant or antiplatelet therapy were generally older and more frequently male. This aligns with the epidemiology of conditions such as atrial fibrillation and coronary artery disease, which are more prevalent in older patients and in men.^16^, ^17^ Although age and male sex are also independent risk factors for urologic malignancies, the tumours in our cohort were detected following episodes of haematuria. This suggests that haematuria, even when occurring under anticoagulant or antiplatelet therapy, may serve as a symptom that unmasks previously undiagnosed malignant disease. In our cohort, the youngest patient diagnosed with a urologic malignancy in the setting of haematuria was 43 years old, which supports maintaining a low threshold for performing cystoscopy, particularly in cases of visible haematuria.

When examining the two largest groups in our study — those taking DOACs and those taking antiplatelet medication — a pronounced difference is observed among the patients taking antiplatelet agents (p = 0.005), but no significant difference was found in the DOACs group (p = 0.689). A possible explanation is a history of smoking, as smokers are more likely to have vascular conditions that require antiplatelet agents and may also develop urologic malignancies, such as bladder tumours, more frequently. However, smoking status was documented for only 69 patients (12.7%), representing approximately one‐eighth of the cohort. This is due to the retrospective design of the study and the fact that, when patients present in the emergency room, most physicians do not routinely inquire about smoking status because of time constraints and prioritization of acute care. Consequently, the sample size is insufficient to perform meaningful statistical analyses regarding smoking status. Patients on DOACs had a higher rate of previous surgeries, which is understandable given that DOACs are associated with more bleeding complications than antiplatelet agents, as antiplatelet agents typically do not need to be discontinued prior to surgery.

Of the 268 patients on anticoagulant or antiplatelet therapy, only 9.3% (n = 25) had no identifiable underlying cause for gross haematuria, suggesting that medication alone was rarely the sole contributor.

No thromboembolic complications were recorded in our cohort.

The results of this study should be interpreted within the limitations of its retrospective design.

## 5 CONCLUSION

It cannot be assumed that patients with haematuria who are on anticoagulant and antiplatelet therapy are bleeding solely due to their medication. Until proven otherwise, the possibility of an underlying tumour must always be considered. Our data indicate that this is particularly true for patients on such medications, underscoring the need for further diagnostic evaluation, regardless of anticoagulant use.

## AUTHOR CONTRIBUTIONS

Study concept and design: Yushan Yang, Johannes Breyer, Markus Resch. Data acquisition: Yushan Yang, Johanna Seidl, Johannes Breyer. Statistical analysis: Yushan Yang, Johanna Seidl, Johannes Breyer, Markus Resch. Manuscript drafting: Yushan Yang, Johanna Seidl, Johannes Breyer, Markus Resch. All authors have contributed to the final version of the manuscript.

## CONFLICT OF INTEREST STATEMENT

The authors declare that they have no conflicts of interest in this study. The authors declare that no funds, grants or other support were received during the preparation of this manuscript.
